# Sulforaphane enhanced muscle growth by promoting lipid oxidation through modulating key signaling pathways

**DOI:** 10.1042/BSR20240084

**Published:** 2024-07-03

**Authors:** Rui Zhang, Suqin Chen, Feng Zhao, Wei Wang, Dayu Liu, Lin Chen, Ting Bai, Zhoulin Wu, Lili Ji, Jiamin Zhang

**Affiliations:** 1 Meat Processing Key Laboratory of Sichuan Province, College of Food and Biological Engineering, Chengdu University, Chengdu, China; 2 Department of Oncology, Sichuan Provincial People's Hospital, School of Medicine, University of Electronic Science and Technology of China, Chengdu, China

**Keywords:** fiber size, lipid, metabolism, muscle growth, protein, Sulforaphane

## Abstract

Sulforaphane (SFN) has shown diverse effects on human health and diseases. SFN was administered daily to C57BL/6J mice at doses of 1 mg/kg (SFN1) and 3 mg/kg (SFN3) for 8 weeks. Both doses of SFN accelerated body weight increment. The cross-sectional area and diameter of *Longissimus dorsi* (LD) muscle fibers were enlarged in SFN3 group. Triglyceride (TG) and total cholesterol (TC) levels in LD muscle were decreased in SFN groups. RNA sequencing results revealed that 2455 and 2318 differentially expressed genes (DEGs) were found in SFN1 and SFN3 groups, respectively. Based on GO enrichment analysis, 754 and 911 enriched GO terms in the SFN1 and SFN3 groups, respectively. KEGG enrichment analysis shown that one KEGG pathway was enriched in the SFN1 group, while six KEGG pathways were enriched in the SFN3 group. The expressions of nine selected DEGs validated with qRT-PCR were in line with the RNA sequencing data. Furthermore, SFN treatment influenced lipid and protein metabolism related pathways including AMPK signaling, fatty acid metabolism signaling, cholesterol metabolism signalling, PPAR signaling, peroxisome signaling, TGFβ signaling, and mTOR signaling. In summary, SFN elevated muscle fibers size and reduced TG and TC content of in LD muscle by modulating protein and lipid metabolism-related signaling pathways.

## Introduction

Sulforaphane (SFN), a naturally isothiocyanate derived from glucoraphanin, has received considerable attention owing to its potential therapeutic applications in last two decades. A amount of work have provided robust evidences of its effects on diverse biological activities, encompassing antioxidant [1], anti-inflammatory [2], and anticancer properties [3]. By acting as a potent inducer of phase II detoxification enzymes, SFN exerts potent chemopreventive effects by bolstering the detoxification and elimination of carcinogens [4]. Furthermore, SFN has demonstrated its capability to modulate multiple signaling pathways implicated in cellular metabolism, including lipid [5], protein [6], and glucose [7]. The multifaceted actions of SFN position it as an appealing bioactive phytochemicals for further exploration due to its potential beneficial effects on various aspects of human health, including skeletal muscle function.

Skeletal muscle is not only responsible for body movement but also plays an essential role in overall metabolism and energy balance. The proper balance of lipid and protein metabolism are essential in the development, maintenance, and overall functioning of skeletal muscles. Muscle wasting results from inadequate nutrition, extended immobilization, advancing age, and other factors. This manifests as a decline in muscle function and structure, which could be attributed to an imbalance in protein synthesis and breakdown related signaling pathway like mammalian target of rapamycin (mTOR) [8], transforming growth factor-β (TGFβ) [9], etc. Disruptions in lipid metabolism related signaling, such as peroxisome proliferator-activated receptors (PPARs) [10], affect the fatty acid β-oxidation in muscles, contributing to the pathogenesis of Type 2 diabetes and metabolic syndrome [11]. Various phytochemicals, such as curcumin and SFN, have been reported to benefit muscle function and mass [12].

SFN shows a promising penitential application in skeletal muscle protection and the recovery from muscle atrophy and damage. SFN treatment attenuated the inflammation and muscular pathology in mice model for muscle atrophy [13,14]. SFN administration extends muscle endurance and protects muscle from exhaustive training through activation of NFE2L2 antioxidant pathway [15,16]. Our previews work also found that SFN augments the skeletal muscle growth by inhibiting the Myostatin/Smad7 signaling pathway [17,18]. However, limited work has focused on the role of SFN in maintain the balance of protein and lipid metabolism in skeletal muscle. The current work investigate the effects of SFN at an everyday consumption level on protein and lipid metabolism in skeletal muscle.

## Materials and methods

### Animals and experimental protocol

A total of twenty-one male SPF C57BL/6J mice, 4 weeks old, were purchased from Chengdu Dossy Experimental Animals Co., LTD. SFN (HY-13755, MedChemExpress) was dissolved in dimethyl sulfoxide (DMSO) and subsequently diluted with phosphate-buffered saline (PBS). Mice were randomly divided into three groups, with seven mice in each group: the control (Ctrl) group, the SFN1 group, and the SFN3 group. In the Ctrl group, the mice received an intraperitoneal (i.p.) injection of DMSO diluted with PBS in the same amount compared with SFN groups. In the SFN1 group, the mice received SFN at a dosage of 1 mg per kilogram of body weight per day (1 mg/kg/d BW) using i.p. injection. In the SFN3 group, the mice received SFN at a dosage of 3 mg/kg/d BW using i.p. injection. The experiment was carried out for 8 weeks and the body weight was measured every week. At the end of the experimental, mice were euthanized by cervical dislocation. *Longissimus dorsi* (LD) muscle samples were collected from the mice, immediately frozen in liquid nitrogen and stored at −80°C for further analysis. All experimental procedures were approved and conducted in strict accordance with the guidelines outlined in the Management Policy for Experimental Animals of Chengdu University.

### Measurements of triglyceride (TG) and total cholesterol (TC)

A 50 mg sample of LD muscle was used for the measurement of TG and TC levels. The assays for TG and TC were performed using E1013 and E1015 kits (Applygen, China) based on the GPO Trinder methodology, respectively. The mean value of two repetitive measurements were got for each sample.

### Histological analysis

The LD muscle samples were fixed in 4% paraformaldehyde for 24 h and subsequently embedded in paraffin for histological analysis. Sections of 5 µm thickness were prepared perpendicular to the muscle fascicles and stained with Hematoxylin-Eosin (H&E) following standard protocols. Images were captured at a magnification of 40× using a visible light microscope (BA210 Digital, Motic, Fujian, China). Motic Images Advanced 3.2 software was employed for image analysis. For each muscle sample, ten measurements of fibre diameter and cross-sectional area were conducted, and the results were reported as mean ± standard deviation (SD).

### RNA sequencing (RNA-seq) and data analysis

Total RNA was isolated using Trizol (Invitrogen, Shanghai, China). RNA quality was assessed on an Agilent 2100 Bioanalyzer (Agilent Technologies, Palo Alto, CA, U.S.A.) and checked using RNase free agarose gel electrophoresis. For RNA-seq analysis, a pooled total RNA sample was generated by combining an equal amount (μg) of mRNA from seven LD muscle for each group. The mRNA was enriched by Oligo(dT) Beads. Then, the enriched mRNA was fragmented into short fragments using fragmentation buffer and reversly transcribed into cDNA by using NEBNext Ultra RNA Library Prep Kit for Illumina (New England Biolabs, MA, U.S.A.). The resulting cDNA library was sequenced using Illumina Novaseq6000 by Gene Denovo Biotechnology Co. (Guangzhou, China).

Raw reads containing adapters or low quality bases were filtered by fastp [19] to get high quality clean reads. The rRNA mapped reads were removed. Short reads alignment tool Bowtie2 [20] was used for mapping reads to ribosome RNA (rRNA) database. An index of the reference genome was built, and paired-end clean reads were mapped to the reference genome using HISAT2. 2.4 [21] and other parameters set as a default. The mapped reads of each sample were assembled by using StringTie v1.3.1 [22] in a reference-based approach. For each transcription region, a FPKM (fragment per kilobase of transcript per million mapped reads) value was calculated to quantify its expression abundance and variations, using RSEM [23] software.

### Differentially expressed genes (DEGs) and bioinformatics analysis

RNAs differential expression analysis was performed by DESeq2 [24] software between two different groups. The genes/transcripts with the parameter of |log2(fold change)| > 1 and false discovery rate (FDR) < 0.05 were considered DEGs.

Gene Ontology (GO) is an international standardized gene functional classification system which offers a dynamic-updated controlled vocabulary and a strictly defined concept to comprehensively describe properties of genes and their products in any organism [25]. GO has three ontologies: molecular function, cellular component and biological process. The basic unit of GO is GO-term. Each GO-term belongs to a type of ontology. GO enrichment analysis provides all GO terms that significantly enriched in DEGs comparing with the genome background, and filter the DEGs that correspond to biological functions. First, all DEGs were mapped to GO terms in the Gene Ontology database (http://www.geneontology.org/), gene numbers were calculated for every term, significantly enriched GO terms in DEGs comparing to the genome background were defined by hypergeometric test. *Q*-value (adjusted *P*-value) < 0.05 as a threshold. GO terms meeting this condition were defined as significantly enriched GO terms.

KEGG is the major public pathway-related database [26]. Pathway-based analysis helps to further understand genes biological functions. Pathway enrichment analysis identified significantly enriched metabolic pathways or signal transduction pathways in DEGs comparing with the whole genome background. The calculated *P*-value was gone through FDR Correction, taking *Q-*value < 0.05 as a threshold. Pathways meeting this condition were defined as significantly enriched pathways in DEGs

### Quantitative real-time PCR (qRT-PCR)

RevertAid™ Master Mix (Thermo Scientific, China) was used to synthesis first stand cDNA. The qRT-PCR was carried out using Platinum SYBR Green qPCR SuperMix-UDG kit (Thermo Fisher Scientific, Inc.). The Primer3 was utilized to design all primers for qRT-PCR [27] and the primer sequences were shown in Table 1. The Ct value from qRT-PCR was analysed using the 2^−ΔΔCt^ method [28]. Gapdh and β-actin were used as endogenous references for mRNA.

**Table 1:** Primer sequences for qRT-PCR verification

Gene	Gene ID	Forward primer (5′→3′)	Reverse primer (5′→3′)	Product (bp)
Acox1	11430	ACACCCACCCACCAAGAAAG	GTCAGGAAGTGGGGTCATGG	96
Acsl1	14081	GGAAGCCAAACCAGCCCTAT	AAGAGGCCGATGAACTGCTC	124
Acadm	11364	TGGTCCTTAGCCCCGAATTG	GTTCTTCCTTGACAAGCCGC	115
Pparα	19013	TCCAGGGTTCAGTCCAGTGT	AGGGACAGTGACAGGTGAGG	125
Npy	109648	GGCTTGAAGACCCTTCCATGT	TAGTGGTGGCATGCATTGGT	129
Acacb	100705	TTTTGCCTGAGGTGGGGATC	CTTGGGTCTCATCTGGCGTT	127
Prkaa2	108079	ATCACACCACCACCAAGCAA	CTCCCAGCTACCCCAGTCT	143
Irs1	16367	GGCCCAGAACATGCATGAGA	GTTGTTGAGATGGTGCCTGC	139
Mapk1	26413	TTCTGCACCGTGACCTCAAG	ATCTGGATCTGCAACACGGG	98
β-actin	11461	GTGGATCAGCAAGCAGGAGT	ACGCAGCTCAGTAACAGTCC	86
Gapdh	14433	ACTGAGCAAGAGAGGCCCTA	GGTGGGTGCAGCGAACTTTA	150

### Statistical analysis

Student’s *t-*test and one-way ANOVA was applied to determine the statistical significance between the control group and SFN treatment groups. The data were expressed as mean ± SD. *P<0.05, **P<0.01, and ***P<0.001 were utilized as levels of significance.

## Results

### Body weight, muscle microstructure, and lipid levels

**Figure 1:**
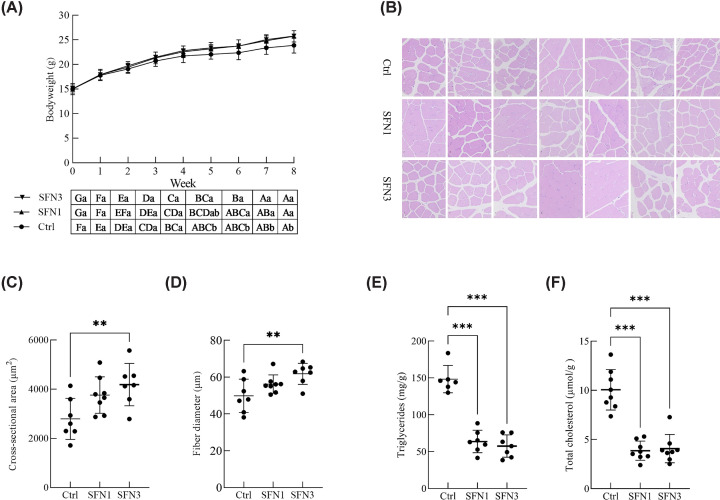
Effect of SFN on muscle size and lipid content in mice (**A**) Body weight of mice over eight weeks and values with different lowercase letters (a, b, and c) at the same week and uppercase letters (A, B, and C) in the same group were significantly different from each other (*P*<0.05). (**B**) H&E staining of LD muscle. (**C,D**) Cross-section area (C) and diameter (D) of muscle fibre. (**E,F**) TG (E) and TC (F) content in LD muscle. Data were shown as the mean ± SD, *n*=7, **P<0.01, ***P<0.001.

Body weight was measured every week. At the fifth week, the body weight of SFN3 group was significant higher than that of Ctrl group. Both SFN3 and SFN1 group maintained the significant higher body weight than that of Ctrl group since the sixth week. However, there was no significant difference between two SFN groups (Figure 1A). After scarification, histological analysis was performed for LD muscle (Figure 1B). The muscle fiber diameter and cross-section area was significantly increased in SFN3 group, not in the SFN1 group (Figure 1C,D). The TG and TC content were measured in LD muscle. It was found that TG and TC content were significantly reduced in LD muscle (Figure 1E,F).

### RNA-seq revealed the transcriptome alterations in LD muscle

**Figure 2:**
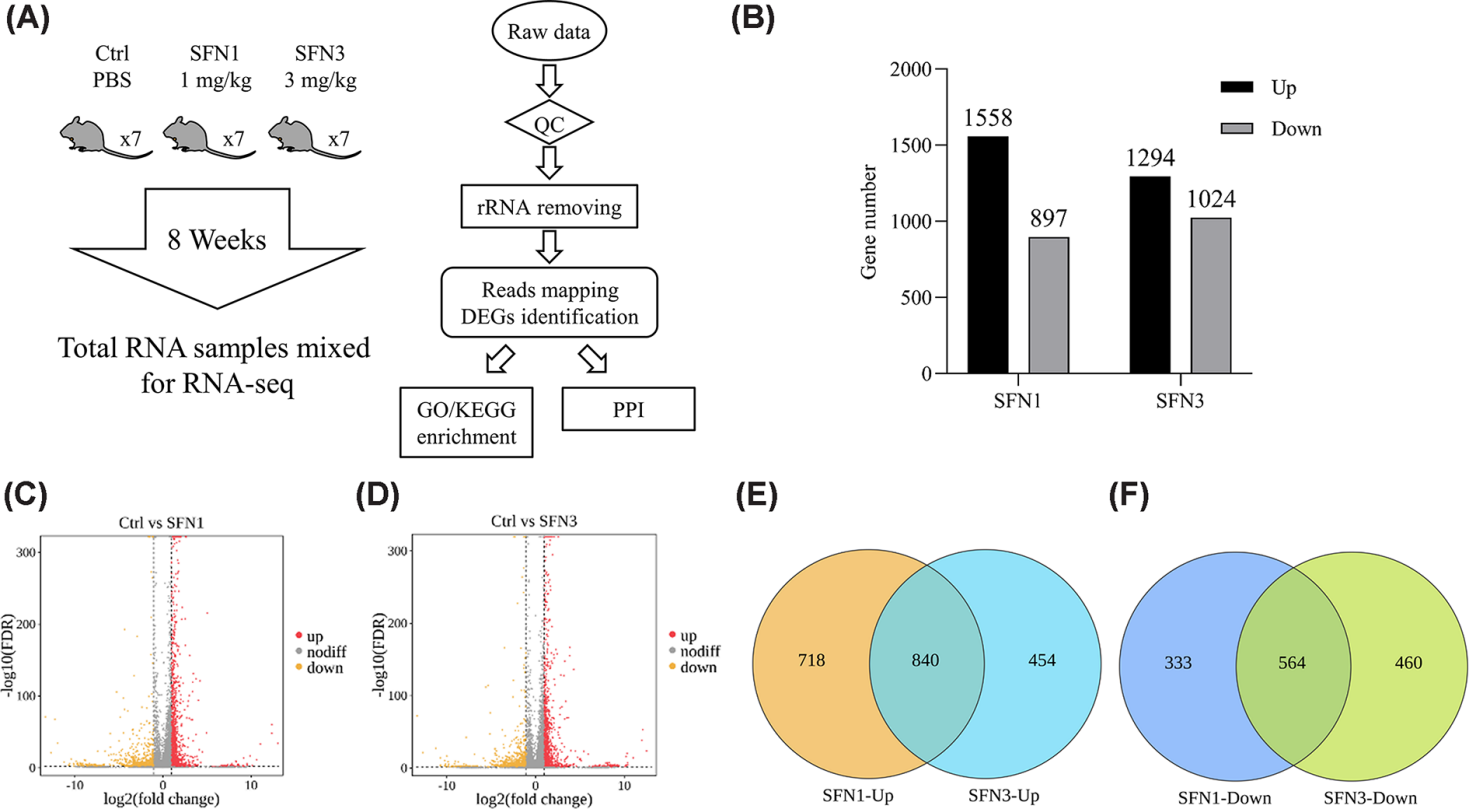
RNA-seq applied in LD muscle (**A**) Work flow for RNA-seq. (**B**) DEGs in SFN1 and SFN3 groups. (**C,D**) Up- and down-regulated DEGs in SFN1 (C) and SFN3 (D) vs. Ctrl group. (**E,F**) Venn diagrams of up-regulated genes (E), and down-regulated genes (F) in SFN1 and SFN3 groups. Data are derived from RNA-seq analysis of one pooled total RNA sample for each group (*n*=1).

To reveal the underling mechanisms, RNA-seq was used to investigate effects of SFN on transcriptome of LD muscle (Figure 2A) and related statistical information was listed in Table 2. Out of sequenced genes, differentially expressed genes (DEGs) was selected based with the criterion at |log2(fold change)| > 1 and FDR < 0.05. 2455 DEGs were found in SFN1 group in contrast to Ctrl group (Figure 2B,C), where 1558 DEGs were up-regulated and 897 DEGs were down-regulated. Similarly, 2318 DEGs was found in SFN3 group in comparison with Ctrl group with 1294 up-regulated DEGs and 1024 down-regulated DEGs (Figure 2B,D). As shown in Figure 2E, 840 DEGs were up-regulated in both SFN groups, while 718 and 454 DEGs were uniquely up-regulated in SFN1 and SFN3, respectively. At the meantime, 564 DEGs were down-regulated in both SFN groups, while 333 and 460 DEGs were uniquely decreased in SFN1 and SFN3 group, respectively (Figure 2F).

**Table 2:** RNA-seq data quality control and statistical information

Samples	Raw data		Quality control				rRNA removing and read mapping		
	Raw data	Clean data (%)	Clean data (bp)	Q20 (%)	Q30 (%)	GC (%)	Clean reads	Unmapped reads (%)	Unique mapped (%)
Ctrl	39410948	39059956 (99.11%)	5792430501	5624717154 (97.10%)	5345829241 (92.29%)	2711861554 (46.82%)	39059956	38551734 (98.70%)	29994495 (77.80%)
SFN1	45553336	45308302 (99.46%)	6745733890	6541638540 (96.97%)	6194594540 (91.83%)	3321648136 (49.24%)	45308302	45121678 (99.59%)	38895978 (86.20%)
SFN3	48919862	48631948 (99.41%)	7234317207	7021661773 (97.06%)	6666549422 (92.15%)	3478417660 (48.08%)	48631948	48322706 (99.36%)	40449061 (83.71%)

### GO and KEGG enrichement analysis

**Figure 3:**
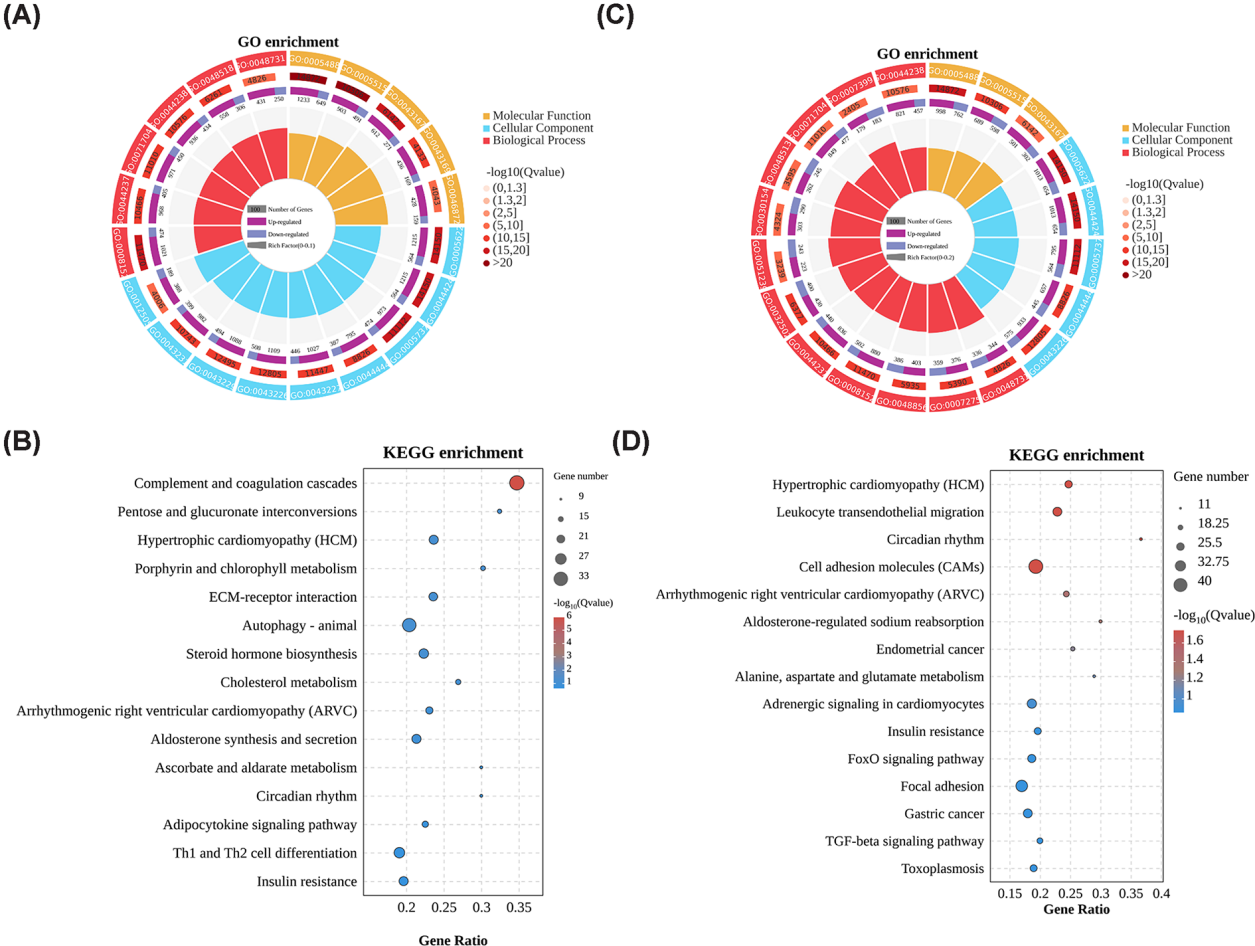
Enrichment analysis of RNA-seq data (**A,B**) GO (A) and KEGG (B) pathway enrichment for DEGs in SFN1 groups. (**C,D**) GO (C) and KEGG (D) pathway enrichment for DEGs in SFN3 groups. Data are derived from RNA-seq analysis of one pooled total RNA sample for each group (*n*=1).

GO and KEGG enrichment analysis was analyzed for DEGs in SFN1 and SFN3 groups. For GO enrichment analysis, 2455 DEGs in SFN1 groups were significantly enriched in 106 cellular component terms, 35 molecular function terms, and 613 biological process terms. Based on *Q*-values, the top ten terms terms in each catogary were shown in Figure 3A. For DEGs in SFN1 groups, Complement and coagulation cascades pathway (ko04610) was significantly enriched (*Q*-value = 9.42E-07). The top ten pathways were shown in Figure 3B.

During GO enrichment analysis, 2496 DEGs in SFN3 groups were significantly enriched in 107 cellular component terms, 66 molecular function terms, and 738 biological process terms (Figure 3C). For KEGG enrichment analysis, the following six pathways were significantly enriched: Hypertrophic cardiomyopathy (ko05410), Leukocyte transendothelial migration (ko04670), Circadian rhythm (ko04710), Cell adhesion molecules (ko04514), Arrhythmogenic right ventricular cardiomyopathy (ko05412), and Aldosterone-regulated sodium reabsorption (ko04960) with a *Q-*value < 0.05 (Figure 3D).

### SFN enhanced lipid metabolism related signaling pathways

**Figure 4:**
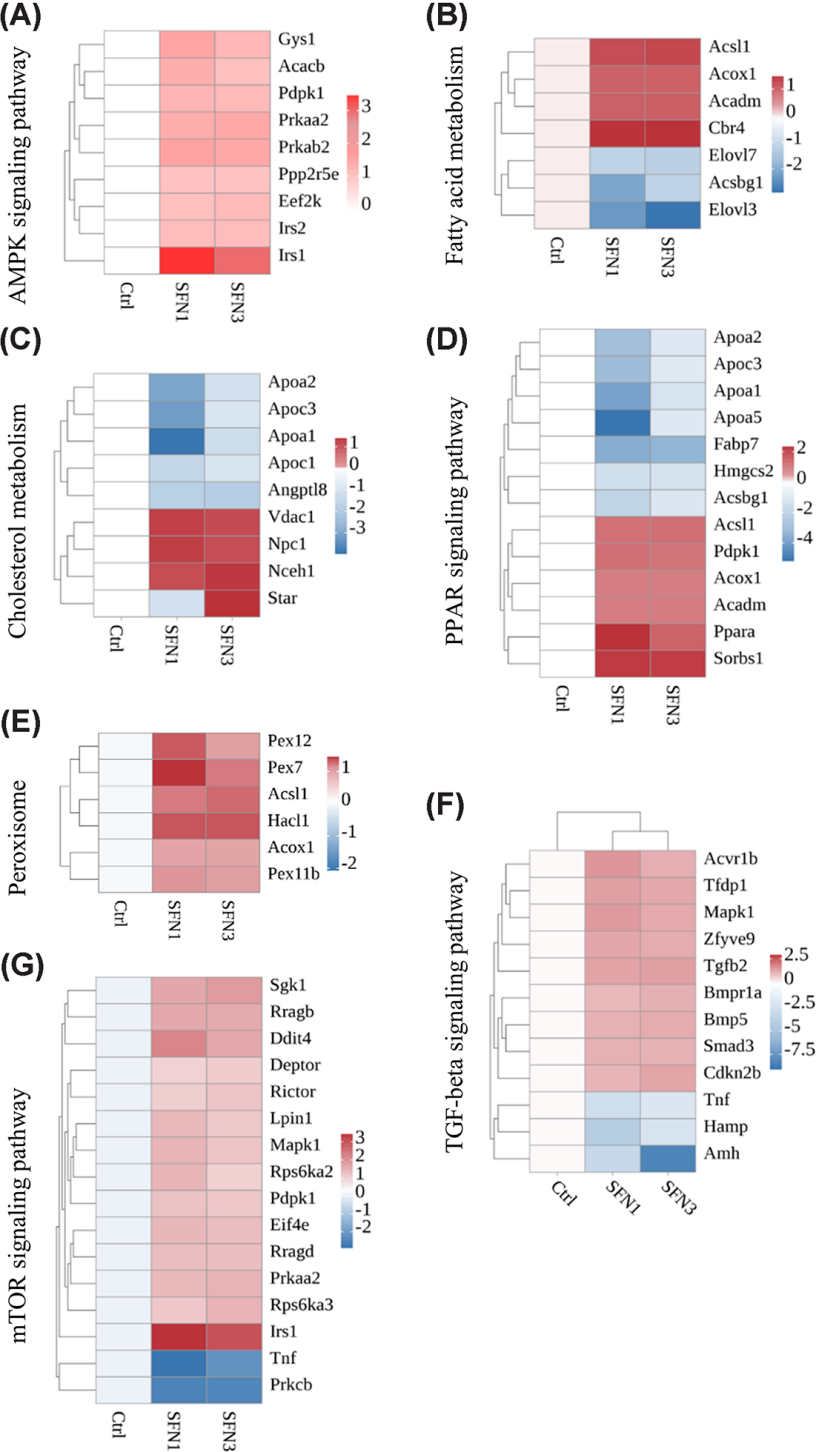
Heatmaps for gene expression in lipid and protein metabolism pathway (**A**) AMPK signaling pathway. (**B**) Fatty acid metabolism. (**C**) Cholesterol metabolism. (**D**) PPAR signaling pathway. (**E**) Peroxisome. (**F**) Peroxisome. (**G**) mTOR signaling pathway. Data are derived from RNA-seq analysis of one pooled total RNA sample for each group (*n*=1).

In order to reveal how SFN regulated the balance of lipid and protein metabolism, the common DEGs in related pathways from both SFN groups was further analyzed. AMP-activated protein kinase (AMPK) signaling is the master regulator of cellular energy balance. After SFN administration, as shown in Figure 4A, AMPK signaling pathway was activated by SFN treatment. The up-regulated Prkaa2 and Prkab2 were in cooperation with the up-regulated Irs1, Irs2, and Ppp2r5e to turn the AMPK signaling pathway on. The Gys1, Acacb, and Eef2k were the key regulator of glycogen, fatty acid, and protein synthesis. Their mRNA expression were up-regulated in both SFN groups.

As the levels of TG and TC were decreased in LD muscle treated with SFN, the fatty acid and cholesterol metabolism related pathways were investigated. As shown in Figure 4B, Acox1, Acadm, and Acsl1 were up-regulated to promote fatty acid β-oxidation. Elvol3 and Elovl7 were downregulated to inhibited fatty acid synthesis. As for cholesterol metabolism shown in Figure 4C, four apolipoprotein genes including Apoa1, Apoa2, Apoc1, and Apoc3 was down-regulated to inhibit the transportation of high density lipoprotein cholesterol. Nceh1, Vdac1, and Npc1 were up-regulated to enhance the degradation of cholesterol.

PPAR signaling pathway is closely linked to lipid uptake, synthesis, and oxidation. Besides four downregulated apolipoprotein genes, Fabp7, Hmgcs2, and Acsbg1 were down-regulated in SFN group to inhibit the fatty acid uptake and synthesis (Figure 4D). Peroxisome is a critical cellular organelle fatty acid metabolism and is responsible for breakdown of very-long-chain and branched-chain fatty acids through β-oxidation into acetyl-CoA molecules utilized for energy production in the mitochondria. As shown in Figure 4E, peroxisomal gene Pex7, Pex11b, and Pex12 were up-regulated to enhance peroxisomal biogenesis and matrix protein import. Acsl1 and Acox1 were up-regulated to promote the fatty acid utilization and oxidation.

### SFN enhanced muscle growth and protein turnover related signaling pathways

As muscle fiber size was significantly increased in SFN3 group, the muscle growth and protein synthesis related signaling pathways were analyzed. As shown in Figure 4F, nine TGFβ signaling pathway related gene including Bmp5, Tgfb2, Bmpr1a, Acvr1b, Smad3, Zfyve9, Cdkn2b, Tfdp1, and Mapk1 were significantly up-regulated in both SFN groups. In addition, 14 genes mTOR signaling pathway including Irs1, Rragb, Rragd, Rictor, Deptor, Eif4e, Sgk1, Ddit4, Mapk1, Rps6ka2, Lpin1, Prkaa2, Pdpk1, and Rps6ka3 were significantly up-regulated to support muscle protein synthesis (Figure 4G).

### qRT-PCR verification of lipid metabolism related genes

**Figure 5:**
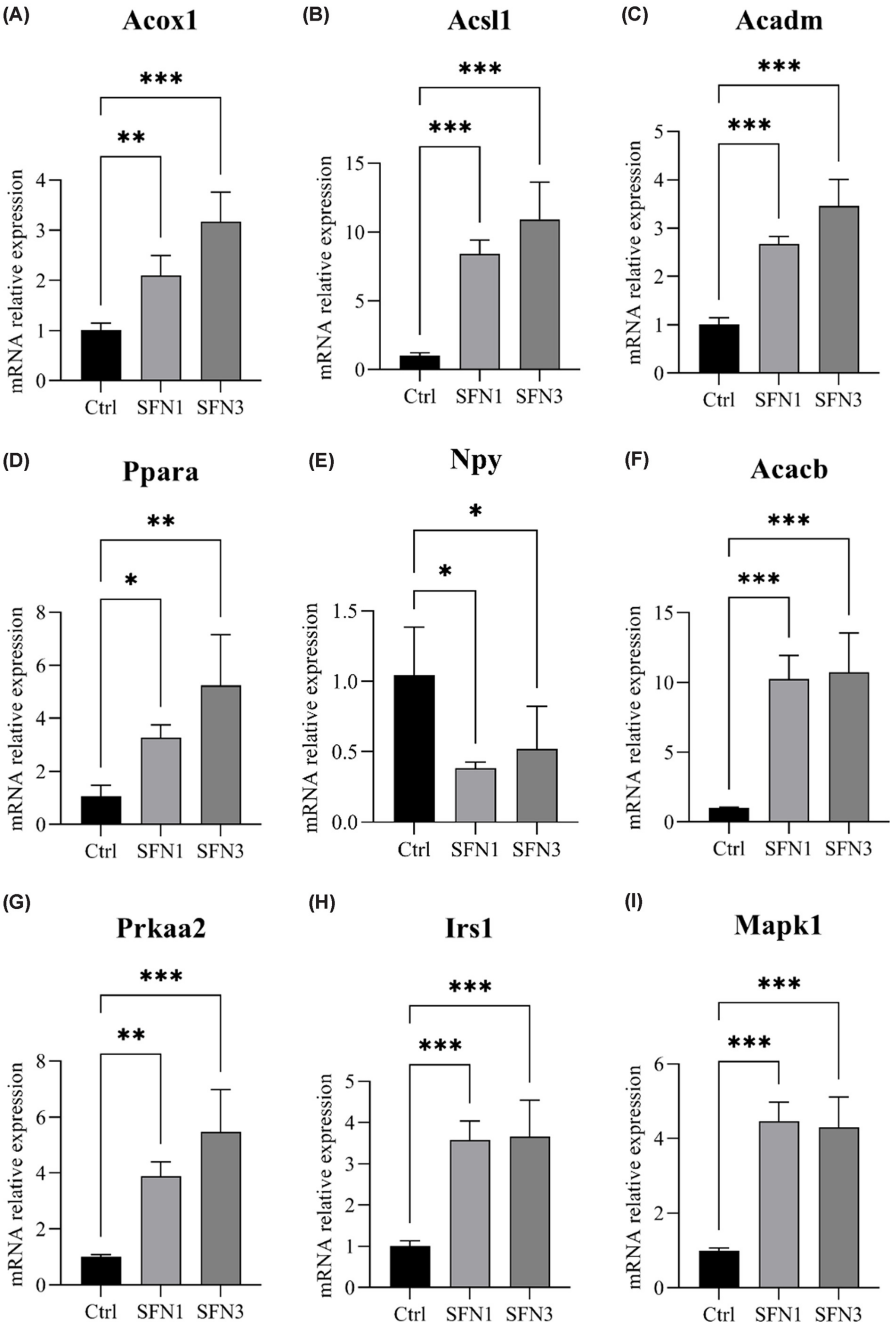
qRT-PCR verification of selected DEGs relative mRNA expression (**A–I**) Acox1, Acsl1, Acadm, Ppara, Npy, Acacb, Prkaa2, Irs1, and Mapk1. *n*=3 and *P<0.05, **P<0.01, ***P<0.001.

In order to verify the RNA-seq results, gene expression of selected DEGs was quantified with qRT-PCR. As shown in Figure 5, qRT-PCR results were in line with RNA-seq data.

### Protein–protein interaction (PPI) analysis for selected DEGs

**Figure 6:**
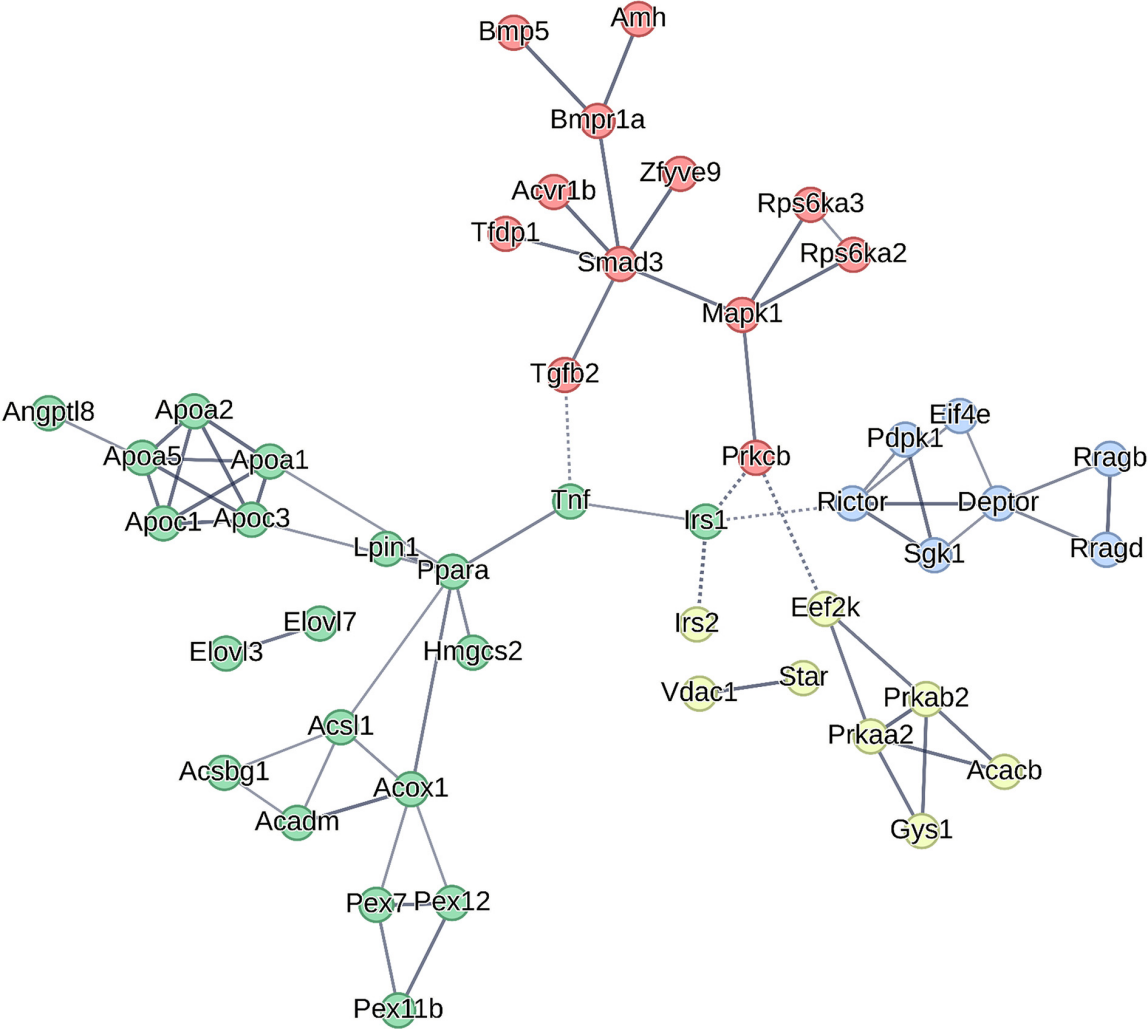
Protein–protein interaction network for DEGs in lipid and protein signaling pathway The nodes mean genes. The edges indicate both functional and physical associations. Distinct colors stand for different clusters.

To elucidate interactions of lipid and protein metabolism related DEGs mentioned above. These DEGs, 57 in total, were submitted to the STRING V12.0 databases to construct a PPI network including both functional and physical protein associations. A minimum required interaction score of 0.7 were applied in PPI calculations for a balance between high quality of interactions and low false-positive ratio. The disconnected nodes were not shown in the network. The PPI network of selected DEGs was consisted of number of 48 nodes and 65 edges with an enrichment *P*-value < 1.0E-16 (Figure 6). The top three node connecting most amount of edges were Ppara (7 edges), Smad3 (6 edges), and Acox1, Apoa5, Apoa1, Apoc3, Deptor, and Rictor (5 edges). Furthermore, PPI network was clustered with *k*-means for four groups in different colors: PPAR signaling in green with 20 nodes, TGFβ signaling in red with 12 nodes, AMPK signaling in yellow with 8 nodes, and mTOR signaling in blue with 7 nodes.

## Discussion and conclusion

SFN has been extensively studied for its role in antioxidant defense and as a chemoprotective agent against tumors. Additionally, SFN has demonstrated effects on various human diseases and health conditions, including diabetes, muscle atrophy, autism, and ophthalmic disease, among others. The present study aims to investigate effects of SFN as a daily dietary supplementation on skeletal muscle and its underlying mechanisms.

SFN is a widely researched bioactive compound derived from cruciferous vegetables, such as broccoli, cabbage, and broccoli sprouts. SFN exerts its effects through two main underlying mechanisms: low-level antioxidation and high-level apoptosis [29]. Similarly, our previous work demonstrated that SFN operates on a dose-dependent model. SFN at 5 and 10 μM can promote the proliferation of skeletal muscle stem cells, while SFN over 10 μM induce cellular apoptosis [17]. Epidemiological studies have concluded that a diet rich in cruciferous vegetables, with servings ranging from 250 to 500 g per day for a duration of 6 to 12 days, can decrease the risk of various types of tumors [29,30]. In animal or cell culture-based studies, SFN concentrations over 5 mg/kg or 10 μM, respectively, were used to investigate the effects of SFN. In these situations, the main effects of SFN are either antioxidation or apoptosis induction.

However, the intake of cruciferous vegetables, like broccoli, is typically restricted to no more than three times a week, with each serving limited to a maximum of 200 g per person. Consuming cruciferous vegetables on this manner may not achieve a substantial enough level to exert a significant influence on human health within a short period. Therefore, investigating SFN at lower levels over an extended period more closely resembles everyday consumption habits. In this study, SFN was administered at doses of 1 and 3 mg/kg, equivalent to concentrations of 0.14 and 0.42 μM, respectively, for a mouse weighing ∼25 g daily to achieve this objective. A similar experiment design was applied to investigate effects of SNF at 2 mg/kg on muscle fibrosis [31].

In our study, we observed a significant acceleration in body weight increment after SFN treatment. This divergence in body weight was initially observed between the Ctrl group and SFN3 group. One week later, the SFN1 group also demonstrated similar patterns to the SFN3 group. However, SFN has been shown to decrease body weight in high-fat diet-fed mice that exhibit insulin resistance [32]. This effect might be primarily attributed to the enhanced burning of lipids. Based on our previous research, we analyzed the skeletal muscle and found that the fiber diameter and cross-sectional area of the LD muscle were significantly larger in the SFN3 groups. This suggests that SFN promotes skeletal muscle hypertrophy, which aligns with our previous work using primary porcine skeletal muscle stem cells [17]. In another study conducted with mdx mice, a model for Duchenne muscular dystrophy, SFN counteracted the decreased body weight observed in the mdx mice and increased the weight of the tibial anterior, extensor digitorum longus, and soleus muscles in these mice [13]. This finding is consistent with the effects of SFN on C2C12 myotubes, where SFN ameliorated dexamethasone-induced muscle atrophy by reducing protein degradation [14]. Therefore, SFN has the potential to enhance skeletal muscle growth by activating muscle stem cells and reducing protein degradation.

Besides adipose tissue and liver, skeletal muscle is another main target of SFN. The insulin resistance of skeletal muscle was relieved by SFN administration with company of activated AMPK and NRF2 signaling pathway [33]. SFN could inhibit TGFβ activity to attenuate muscle fibrosis [34]. Our work found that SFN up-regulated mRNA expressions of genes, including Bmp5, Bmpr1a, and Acvr1b, in the BMP signaling pathway. Both TGFβ and BMP are belonging to TGFβ superfamily. However, they have opposite effects on muscle growth and muscle mass. BMP binds to Bmpr1a to phosphorylate Smad4 and promote muscle growth and Smad4 knockout leads to muscle atrophy. Thus, SFN could also enhance muscle growth through activating BMP signaling.

mTOR is an important regulator in protein synthesis and play a critical role in muscle mass maintain. mTORC1, instead of mTORC2, is critical for muscle mass and function maintains [35]. Here, we found that SFN up-regulate components for both mTORC1 and mTORC2 complex, including Deptor, Rictor, and the downstream effectors, like Lipin1, Elf4e, and Sgk1. RagA/B (GTP)-RagC/D(GDP) is the active form of Rag GTPase, which modulates the location and activity of mTORC1. Rps6ka2 and Rps6ka3 encodes serine/threonine kinases modulating mTOR signaling through phosphorylating RPS6 and EIF4B in mRNA translation [36]. Furthermore, elF4E was also up-regulated by SFN to increase translation efficiency. However, the research on the effects of SFN on mTOR signaling has been in contradictory. Most of tumor based work has reported that SFN suppresses mTOR signaling and results in cellular apoptosis [37,38]. In contrast, SFN can effectively restore the rotenone-attenuated mTOR signaling in striatum [39]. Thus, further work needs to investigate more detail on the effects of SFN on mTOR signaling.

In the DEGs of the SFN1 groups, only the complement and coagulation cascades pathway was found to be significantly enriched. This pathway plays a crucial role in coordinating immune responses and maintaining hemostasis. Interestingly, the complement and coagulation cascades pathway has also been found to be enriched in sucrose-induced muscle atrophy treated with a phytochemical-rich herbal formula called ATG-125 [40]. This finding suggests that SFN may share a similar mechanism with ATG-125 in alleviating muscle atrophy. For the SFN3 group, six KEGG pathways were significantly enriched, and all of these pathways are associated with muscle function and diseases. Multiple studies has reported the benefit effects of SFN on cardiomyopathy [41,42] and regulate circadian rhythms related gene expression [43].

Besides regulating protein balance, SFN has also been shown to play a role in lipid metabolism in various tissues, including liver [7], adipose tissue [44], and kidney [45]. However, there has been limited research investigating the effects of SFN on lipid metabolism in skeletal muscle. The present study reported, for the first time, that SFN administration increased the activity of peroxisomes and enhanced the peroxisomal protein shuttle, which supports enhanced peroxisomal fatty acid β-oxidation. Additionally, the levels of TG and TC in the LD muscle were found to be decreased in both SFN groups. RNA-seq results demonstrated the activation of the AMPK signaling pathway, indicating that SFN may promote energy expenditure. Similarly, SFN has been found to inhibit the decrement of AMPK phosphorylation levels and reduce lipid accumulation in the liver of mice fed a high-fat diet [46].

Fatty acid β-oxidation is the predominant pathway for the fatty acids degradation to produce energy. ACSL1 is responsible for fatty acid utilization and catalyzes the formation of fatty acyl-CoAs for β-oxidation. Knocking out Acsl1 results in a significant decrease of 50–90% in fatty acid oxidation in adipose tissue [47]. Peroxisomal β-oxidation is responsible for the degradation of very-long-chain and branched-chain fatty acids, while short, medium, and most long-chain fatty acids are primarily oxidized in the mitochondria [48]. The mRNA expression of peroxisome genes Pex7, Pex11, and Pex12b was up-regulated in both SFN groups. Pex7 acts as a receptor that imports matrix proteins into the peroxisome, and one of its main substrates is 3-ketoacyl-CoA thiolase, the enzyme that catalyzes the final reaction of peroxisomal fatty acid β-oxidation [49]. Pex12b removes matrix protein receptors from the peroxisome through ubiquitination of its substrates like Pex7. The Pex11 gene family is highly conserved and regulates peroxisome biogenesis [50]. Acox1, the first and rate-limiting enzyme of the peroxisomal β-oxidation pathway, was up-regulated in the SFN groups, indicating an increased capacity for fatty acid β-oxidation. Additionally, the Acadm gene codes for a protein called medium-chain acyl-CoA dehydrogenase, which is essential for fatty acid oxidation and is located in the mitochondria. SFN has been reported to promote fatty acid β-oxidation in mitochondria by activating carnitine palmitoyltransferase 1A in human prostate cancer cells [51]. Here, SFN might also promote mitochondrial β-oxidation by up-regulating the expression of the Acadm gene. Conversely, SFN inhibits fatty acid synthesis by decreasing the expression of the elongation of very long-chain fatty acids-related enzymes Elovl3 and Elovl7.

In summary, SFN administration in an everyday consumption level is able to enlarge the muscle fibre size and reduce the lipid content of LD muscle in mice. SFN redirects the flux of fatty acid to be utilized through β-oxidation in peroxisome and mitochondrial to support muscle growth.

## Data Availability

The processed data required for interpretation of our results are provided within the manuscript. Raw data will be made available upon request. The RNA-seq data reported in this study have been deposited in the China National GeneBank (https://db.cngb.org) with the accession number CNP0005235.

## Abbreviations

AMPK: AMP-activated protein kinase
DEG: differentially expressed gene
DMSO: dimethyl sulfoxide
GO: Gene Ontology
H&E: Hematoxylin-Eosin
mTOR: mammalian target of rapamycin
PPAR: peroxisome proliferator-activated receptor
PPI: protein–protein interaction
SFN: sulforaphane
TGFβ: transforming growth factor-β
